# Microextrusion Printing of Multilayer Hierarchically Organized Planar Nanostructures Based on NiO, (CeO_2_)_0.8_(Sm_2_O_3_)_0.2_ and La_0.6_Sr_0.4_Co_0.2_Fe_0.8_O_3−δ_

**DOI:** 10.3390/mi14010003

**Published:** 2022-12-20

**Authors:** Tatiana L. Simonenko, Nikolay P. Simonenko, Philipp Yu. Gorobtsov, Elizaveta P. Simonenko, Nikolay T. Kuznetsov

**Affiliations:** Kurnakov Institute of General and Inorganic Chemistry of the Russian Academy of Sciences, 31 Leninsky pr., Moscow 119991, Russia

**Keywords:** hydrothermal synthesis, glycol–citrate synthesis, hierarchical structures, microextrusion printing, functional layer, LSCF, SDC, NiO

## Abstract

In this paper, NiO, La_0.6_Sr_0.4_Co_0.2_Fe_0.8_O_3-δ_ (LSCF) and (CeO_2_)_0.8_(Sm_2_O_3_)_0.2_ (SDC) nanopowders with different microstructures were obtained using hydrothermal and glycol–citrate methods. The microstructural features of the powders were examined using scanning electron microscopy (SEM). The obtained oxide powders were used to form functional inks for the sequential microextrusion printing of NiO-SDC, SDC and LSCF-SDC coatings with resulting three-layer structures of (NiO-SDC)/SDC/(LSCF-SDC) composition. The crystal structures of these layers were studied using an X-ray diffraction analysis, and the microstructures were studied using atomic force microscopy. Scanning capacitance microscopy was employed to build maps of capacitance gradient distribution over the surface of the oxide layers, and Kelvin probe force microscopy was utilized to map surface potential distribution and to estimate the work function values of the studied oxide layers. Using SEM and an energy-dispersive X-ray microanalysis, the cross-sectional area of the formed three-layer structure was analyzed—the interfacial boundary and the chemical element distribution over the surface of the cross-section were investigated. Using impedance spectroscopy, the temperature dependence of the electrical conductivity was also determined for the printed three-layer nanostructure.

## 1. Introduction

The development of advanced “green” power generation technologies in the framework of the constantly increasing energy consumption level and anthropogenic impact on the environment is currently one of the most important tasks of materials science. The decarbonization of the economy has increased the interest of many countries in hydrogen energy devices, in particular, solid oxide fuel cells (SOFCs) [1,2,3,4]. This type of electrochemical generator allows for the direct electrocatalytic conversion of fuel into electricity and heat with high efficiency and extremely low emissions. The applications of such fuel cells are diverse: aerospace, the automotive industry, small and large power plants, portable power generators, combined heat power engineering and reserve power supplies [5,6]. Nevertheless, the widespread introduction and mass commercialization of classical SOFCs are largely limited by their high operating temperatures, which impose stringent requirements for the structural and functional materials used in the fuel cell, accelerating the degradation of the power properties of the device as a whole, thereby increasing the cost of the electricity generated [7,8]. There are two different ways of solving this problem: searching for new materials whose performance in the intermediate-temperature range is not inferior to that of high-temperature materials and reducing the thickness of the SOFC’s main components (electrodes and electrolyte) in order to minimize the total resistance of the device [9,10]. One of the most frequently used compositions of intermediate-temperature electrolyte materials is solid solutions based on cerium dioxide doped with rare-earth oxides [11,12,13]. When considering electrode materials compatible with CeO_2_-based electrolytes in terms of their mutual chemical inertness and thermophysical characteristics, composite cathode materials are usually chosen, representing a mixture of perovskite oxides based on lanthanum cobaltite containing strontium and iron (La_1-x_Sr_x_Co_y_Fe_1-y_O_3-δ_), together with an electrolyte [14,15,16], as well as anode materials containing nickel or its oxide, combined with a solid electrolyte [17,18]. When searching for the optimal composition of cathode and anode materials, not only is the aim to reach a compromise between the linear thermal expansion coefficient and the value of the oxygen-ion conductivity of the electrodes, but it is also to reduce their polarization resistance and to promote an oxygen reduction reaction (ORR) on the cathode, as well as a hydrogen oxidation reaction (HOR) on the anode. It has been reported in a number of works that a cathode containing 50 wt% La_0.6_Sr_0.4_Co_0.2_Fe_0.8_O_3-δ_ and 50 wt% (CeO_2_)_0.8_(Sm_2_O_3_)_0.2_ is characterized by restraining electrode grain growth and the formation of a microstructure that facilitates diffusion and surface exchange, as well as a lower total polarization resistance in comparison with similar cathodes [19,20]. According to the literature [21,22], the ratio of 50 wt% NiO to 50 wt% (CeO_2_)_0.8_(Sm_2_O_3_)_0.2_ is one of the classic variants for anode or anode functional layer formation since this material can have a more developed triple-phase boundary surface (the area where NiO, SDC and hydrogen phases contact), which has a positive effect on HOR efficiency. In addition, this composition demonstrates sufficiently low resistance values and long-term stability.

It is known that the formation of planar devices for microelectronics and alternative energy is associated with high energy costs, when many stages of application and the processing of functional layers are carried out [23]. At the same time, the use of additive manufacturing methods for the fabrication of ceramic materials can significantly improve reproducibility, promote miniaturization (particularly in the context of μ-SOFC [24,25]) and reduce the number of production stages in the automated formation of functional coatings of the desired geometry. Currently, a number of printing technologies are used to produce planar components for alternative energy devices: inkjet printing [26,27,28,29], aerosol printing [30,31,32], screen printing [33,34,35,36], microplotter printing [37,38,39] and microextrusion printing [40,41,42,43,44]. It is worth noting the microextrusion printing method separately due to it having significantly less stringent requirements for the viscosity level of the ink used (compared, for example, with classical inkjet printing), as well as an advantage when forming thick films (the ability to quickly achieve the desired coating thickness when one or a few layers are applied) of the required geometry.

The microstructure of the functional layers is also an important factor that seriously affects the final characteristics of the entire fuel cell. When developing SOFC components with different geometries, researchers often aim to obtain powders with high dispersity, a narrow particle-size distribution and a hierarchical particle organization or ordered porous structure, which generally allows for the formation of materials with a developed surface, facilitates charge transfer and contributes to improved mass transport [45,46]. One of the most convenient approaches towards obtaining materials based on doped cerium dioxide and nickel oxide is the hydrothermal method, which allows for the fine tuning of the microstructural parameters (dispersity, particle shape, porosity level, the degree of crystallinity, etc.) of the resulting product (both in the form of powders and coatings) depending on the objectives to be achieved [47,48,49,50,51,52,53,54]. For oxides of complex composition based on lanthanum cobaltite doped with strontium and iron, combustion methods (glycol–citrate, citrate–nitrate, glycine–nitrate, etc.) are the most suitable way to synthesize them, corresponding to the specific chemical nature of their constituent oxides [16,55,56].

Thus, the purpose of this work was to develop an approach for the additive formation of multilayer hierarchically organized planar nanostructures based on oxides of the composition NiO, (CeO_2_)_0.8_(Sm_2_O_3_)_0.2_ and La_0.6_Sr_0.4_Co_0.2_Fe_0.8_O_3−δ_ on various types of substrates.

## 2. Materials and Methods

### 2.1. Preparation of La_0.6_Sr_0.4_Co_0.2_Fe_0.8_O_3-δ_, (CeO_2_)_0.8_(Sm_2_O_3_)_0.2_ and NiO Nanopowders

Nanopowders of La_0.6_Sr_0.4_Co_0.2_Fe_0.8_O_3−δ_ (LSCF), (CeO_2_)_0.8_(Sm_2_O_3_)_0.2_ (SDC) and NiO composition were obtained using glycol–citrate (LSCF) and hydrothermal synthesis (SDC and NiO) similarly to the methods we described earlier [43,44,57]. In this case, more concentrated (0.3 mol/L) solutions of metal-containing reagents were used to synthesize SDC and NiO. In order to obtain the oxides of the indicated composition, the following reagents were used: La(NO_3_)_3_*6H_2_O (99.99%, LANHIT, Moscow, Russia), Sr(NO_3_)_2_ (>98%, Profsnab, St. Petersburg, Russia), Co(NO_3_)_2_*6H_2_O (>98%, Lenreactiv, St. Petersburg, Russia), FeCl_3_*6H_2_O (>98%, Lenreactiv, St. Petersburg, Russia), Ce(NO_3_)_3_·6H_2_O (99.99%, LANHIT, Moscow, Russia), Sm(NO_3_)_3_·6H_2_O (99.99%, LANHIT, Moscow, Russia), [Ni(C_5_H_7_O_2_)_2_] (>98%, RUSHIM, Moscow, Russia), (NH_2_)_2_CO (99%, RUSHIM, Moscow, Russia) and n-butanol (C_4_H_10_O, 99%, Ekos-1, Moscow, Russia).

### 2.2. Microextrusion Printing of LSCF-SDC, SDC and NiO-SDC Functional Layers

The nanopowders obtained were further used to prepare functional inks for the microextrusion printing of the corresponding LSCF-SDC (ω(SDC) = 50%), SDC and NiO-SDC (ω(SDC) = 50%) coatings. The optimal rheological characteristics of the inks providing the necessary stability and wettability of the polycrystalline Al_2_O_3_ substrate and the Pt/Al_2_O_3_/Pt chip used, as well as maintaining the geometry of the formed oxide coatings, were achieved by using α-terpineol (>97%, Vekton, St. Petersburg, Russia) as a solvent and 20 wt% ethylcellulose (48.0–49.5% (*w*/*w*) ethoxyl base, Sigma Aldrich, St. Louis, MO, USA) as a binder. The optimum mass fraction of the solid-phase particles during the formation of disperse systems based on the (CeO_2_)_0.80_(Sm_2_O_3_)_0.20_ powder was 25%, and in the case of the LSCF-SDC and NiO-SDC electrode materials, the total mass fraction of the oxide particles in the composite functional inks obtained was 15%. The features of the methodology used for the microextrusion printing of oxide coatings have been discussed in our previous papers [43,44]. In the present study, the microextrusion printing of each functional layer was performed sequentially on the surface of a specialized Pt/Al_2_O_3_/Pt chip (Ra = 100 nm, geometric dimensions 4.1 × 25.5 × 0.6 mm; Pt interdigitated electrodes are located on the front side of the Al_2_O_3_ substrate, and the Pt micro-heater is on the reverse side) to evaluate their local electrophysical characteristics with Kelvin probe force microscopy (KPFM) and scanning capacitance microscopy (SCM). A three-layer planar structure of (NiO-SDC)/SDC/(LSCF-SDC) composition was also formed via the layer-by-layer printing of a composite anode, a solid electrolyte and a composite cathode (with their certain displacement relative to each other in the lateral plane in order to avoid the direct contact of electrode materials) on the surface of a polycrystalline Al_2_O_3_ substrate (α- and γ-Al_2_O_3_ phase mixture). After the printing process was completed, each layer was dried (40 °C, 5 h), followed by additional heat treatment (600 °C, 1 h) to remove the residual solvent and to decompose the organic constituents of the functional inks.

### 2.3. Investigation of the Obtained Samples

An X-ray diffraction analysis (XRD) of the obtained planar materials was performed on a Bruker D8 Advance diffractometer (CuKα = 1.5418 Å, Ni-filter, E = 40 kV, I = 40 mA, 2θ range—20–80° and 0.02° resolution, and signal accumulation time per point was 0.3 s).

A cross-section of the (NiO-SDC)/SDC/(LSCF-SDC) 3-layer structure was made after its fixation in epoxy resin using a diamond-coated cutting wheel (followed by the polishing of the examined area). The surface microstructures of the oxide powders and coatings obtained, as well as the cross section of the 3-layer structure, were examined using scanning electron microscopy (Carl Zeiss NVision-40, Carl Zeiss, Inc., Oberkochen, Germany). The chemical compositions of the powders and coatings, as well as the mapping of the chemical element distribution across the cross-sectional area of the sample, were analyzed using an energy-dispersive X-ray microanalysis (energy-dispersive X-ray (EDX) spectrometer INCA X-MAX 80, Oxford Instruments, Abingone, UK).

The topography and local electrophysical properties of the oxide coatings applied sequentially to the surface of the Pt/Al_2_O_3_/Pt chip were additionally studied using atomic force microscopy (AFM), including KPFM and SCM. An NT-MDT Solver PRO-M microscope (NT-MDT, Zelenograd, Russia) and ETALON HA_HR probes (ScanSens, Bremen, Germany) with a W_2_C conductive coating (rounding radius < 35 nm) were used for this purpose. In the course of the KPFM measurements, the work function of the coatings’ surfaces (ϕ_film_) was evaluated. For this purpose, their surface was scanned using a probe with a known output work (ϕ_tip_), the average value of the contact potential (ϕ_CPD_) was calculated for the area scanned, and then the value of ϕ_film_ was determined as the difference between ϕ_tip_ and ϕ_CPD_.

The electrical conductivity of the (NiO-SDC)/SDC/(LSCF-SDC) three-layer planar structure printed on the surface of the Al_2_O_3_ substrate was measured using impedance spectroscopy utilizing an electrochemical test system based on potentiostat/halvanostat P-45X with the impedance measurement module FRA-24M (Electrochemical Instruments, Chernogolovka, Russia). The registration of the impedance hodographs was carried out in a frequency range of 1 MHz–0.1 Hz in the air in a temperature range of 150–600 °C. The sample was heated in a cell placed in a Miterm-12 tube furnace (Microinstrument, Moscow, Russia) with an accuracy of ±1° (the heating accuracy was ensured by the Microinstrument controller MicroCont 1010, Moscow, Russia). The electrical resistance values of the planar samples were calculated from the obtained electrochemical impedance spectroscopy data using ZView software (Scribner Associates Inc., Version3.3c, Southern Pines, NC, USA).

## 3. Results and Discussion

**Figure 1:**
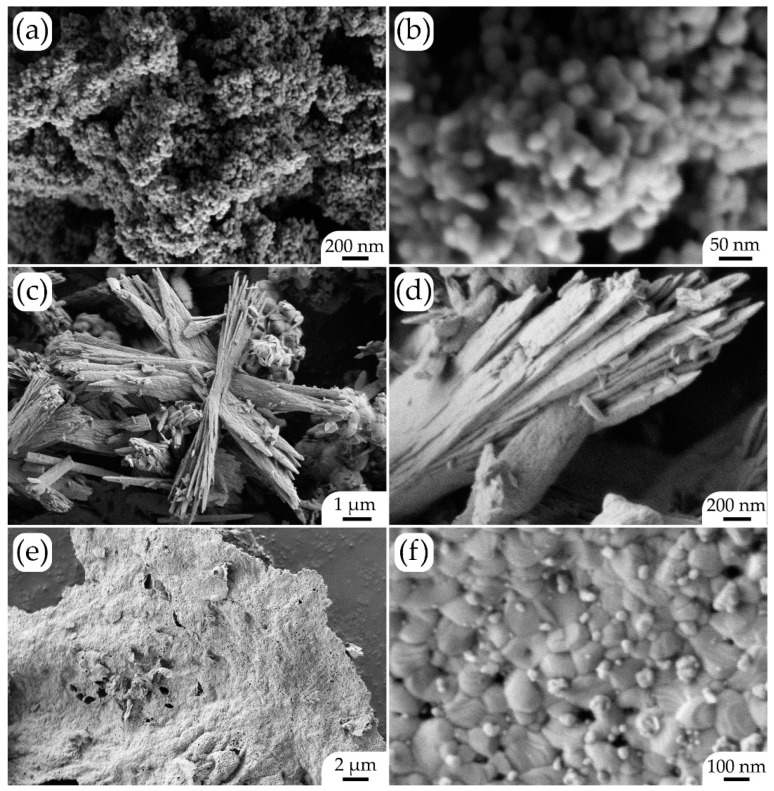
Microstructure of NiO (**a**,**b**), (CeO_2_)_0.8_(Sm_2_O_3_)_0.2_ (**c**,**d**) and La_0.6_Sr_0.4_Co_0.2_Fe_0.8_O_3–δ_ (**e**,**f**) powders, used in the preparation of functional ink for microextrusion printing of oxide coatings.

The microstructure of the obtained oxide powders was studied using scanning electron microscopy (Figure 1). As can be seen in the micrographs (Figure 1a,b), the resulting nickel(II) oxide powder consisted of aggregated spherical nanoparticles with a size of 25 ± 3 nm. No impurities differing from the basic substance in shape or particle size were detected in the powder composition.

In the case of the (CeO_2_)_0.8_(Sm_2_O_3_)_0.2_ solid solution, the powder was characterized by a multilevel hierarchical organization of the particles (Figure 1c,d). Thus, the corresponding nanoscale isotropic particles were ordered into one-dimensional structures about 5 μm long and 200–300 nm wide, united into bundles, which, in turn, were coordinated at an angle of about 60° to each other, forming six-point structures. Besides the above formations, the structure of this powder also contained smaller nanosheets—about 500 nm long, 200–250 nm wide and about 50 nm thick. Thus, under hydrothermal conditions, samarium-doped cerium dioxide with a developed surface was obtained, which also frequently provides a change in the functional characteristics of the material. When synthesizing La_0.6_Sr_0.4_Co_0.2_Fe_0.8_O_3–δ_ oxide, due to large differences in the chemical properties of its constituent metals, the glycol–citrate method was used, which, in such cases, is the optimal approach to obtaining a single-phase nanomaterial of complex composition. The microstructure of the obtained oxide powder is representative of the products synthesized when using this method. As can be seen in the SEM images (Figure 1e,f), the material, due to the foaming of the reaction system, consisted of two-dimensional formations with lateral sizes up to tens of micrometers and thicknesses close to the average particle size. In our case, there was a bimodal distribution of particle sizes—in general, the films consisted of faceted grains sized 120 ± 15 nm, with smaller particles (sized about 20 ± 4 nm) on the surface. Thus, using hydrothermal and glycol–citrate methods, nanoscale oxides of the composition NiO, (CeO_2_)_0.8_(Sm_2_O_3_)_0.2_ and La_0.6_Sr_0.4_Co_0.2_Fe_0.8_O_3−δ_ were synthesized with different microstructures—0D, 1D and 2D, respectively.

**Figure 2:**
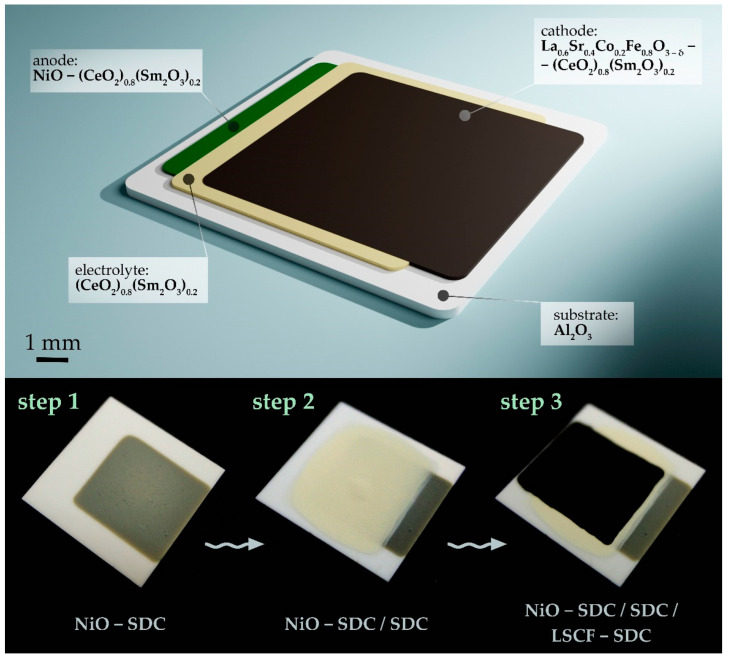
Scheme of the (NiO-SDC)/SDC/(LSCF-SDC) three-layer structure formed (**top**) and the appearance of the functional layers (**bottom**) sequentially deposited on the Al_2_O_3_ substrate surface (left—composite anode layer, middle—electrolyte layer on the anode surface, right—composite cathode layer on the electrolyte surface).

Using functional inks based on the obtained oxide nanopowders, NiO-SDC (the composite anode), SDC (the solid electrolyte) and LSCF-SDC (the composite cathode) coatings were sequentially applied to the surface of the specialized Pt/Al_2_O_3_/Pt chip by using microextrusion printing. Additionally, a (NiO-SDC)/SDC/(LSCF-SDC) three-layer structure was also fabricated on the Al_2_O_3_ substrate surface by using the same method according to the scheme of the functional layer arrangement (Figure 2 top). The appearance of the sample after applying each functional layer (Figure 2 bottom) confirms that the employed technique makes it possible to form both single- and multi-layer functional semiconductor oxide coatings with different chemical compositions, ensuring the targeting of the material and the specified geometry of the resulting planar nanostructures.

**Figure 3:**
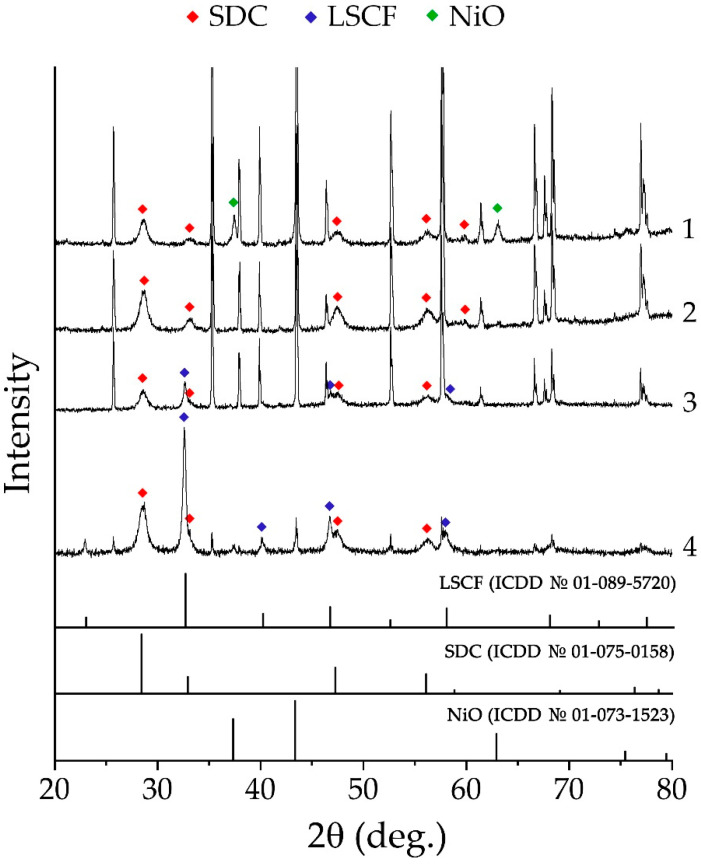
XRD patterns of single- ((NiO-SDC; 1), two- ((NiO-SDC)/SDC; 2), and three-layer ((NiO-SDC)/SDC/(LSCF-SDC); 3 and 4) coatings printed on the surface of Pt/Al_2_O_3_/Pt chip (1–3) and Al_2_O_3_ substrate (4). The unmarked reflections refer to the material of the corresponding substrate.

The crystal structures of the coatings printed on the surfaces of substrates of different types were controlled by using the X-ray diffraction analysis (Figure 3). As can be seen in the X-ray diffraction patterns for the composite anodic coating (NiO-SDC) (Figure 3, sample 1), no chemical interaction between its components and the substrate material occurred during heat treatment—there were reflexes for all the corresponding constituents, while the nickel oxide retained the cubic crystal structure of the halite type (sp.gr. *Fm-3m*; the average size of the coherent scattering region (CSR) was 23 ± 2 nm), and for cerium dioxide, doped with samarium oxide, a cubic structure of the fluorite type was observed (sp.gr. *Fm-3m*; the average size of CSR is 11 ± 1 nm). The analysis of the two-layer coating of the composition (NiO-SDC)/SDC (Figure 3, sample 2) showed that, besides the reflexes of the Pt/Al_2_O_3_/Pt substrate, only the signals of the (CeO_2_)_0.80_(Sm_2_O_3_)_0.20_ electrolyte coating (average OCD size—11 ± 1 nm) were present, which confirms the complete overlapping of the composite electrode layer located below. The three-layer (NiO-SDC)/SDC/(LSCF-SDC) coatings applied to the surfaces of the specialized Pt/Al_2_O_3_/Pt chip and the Al_2_O_3_ substrate (Figure 3, samples 3 and 4, respectively) also only showed a signal from the upper functional layer, the composite cathode, in the corresponding XRD patterns. The type of crystal lattice and the value of the average CSR size for the components of this composite coating conform to the used powders of the corresponding composition (La_0.6_Sr_0.4_Co_0.2_Fe_0.8_O_3−δ_ has a perovskite-type cubic crystal structure, sp.gr. *Pm-3m*, and the average SCR size was 28 ± 3 nm; in the case of the (CeO_2_)_0.80_(Sm_2_O_3_)_0.20_ oxide, the SCR size corresponded to the aforementioned value).

**Figure 4:**
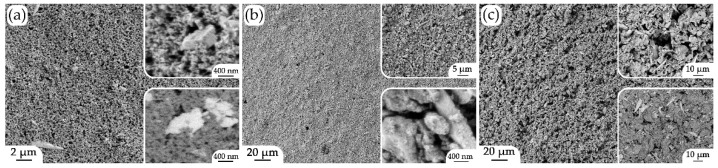
Surface microstructure of single- (**a**), two- (**b**) and three-layer (**c**) coatings deposited sequentially on an Al_2_O_3_ substrate: (**a**) NiO-SDC (top inset—secondary electron detector; bottom inset—reflected electron detector); (**b**) (NiO-SDC)/SDC; (**c**) (NiO-SDC)/SDC/(LSCF-SDC) (top inset—secondary electron detector; bottom inset—reflected electron detector).

The microstructures of the oxide coatings applied sequentially to the surface of the Al_2_O_3_ substrate were studied using scanning electron microscopy (Figure 4). According to the SEM images, the NiO-SDC composite coating surface (Figure 4a) was porous and nanodispersed. The surface examination with the simultaneous use of secondary and backscattered electron detectors allowed for the presence of two phases in the composition of the studied material to be observed in the average atomic number distribution mode, and their target composition was confirmed using an energy-dispersive X-ray microanalysis. The analysis of the (NiO-SDC)/SDC two-layer coating (Figure 4b), where the upper layer was a (CeO_2_)_0.80_(Sm_2_O_3_)_0.20_ solid electrolyte, showed that its microstructure was denser and that the material surface was characterized by the presence of anisotropic oxide particles, whose size and shape agree with the features of the powder used to prepare the corresponding functional inks. When studying the surface of the three-layer structure (Figure 4c), where the upper layer had the LSCF-SDC composition (the presence of the two corresponding phases was evident in the mode of the average atomic number distribution using the backscattered electron detector), an increased porosity, which is an important characteristic of solid oxide fuel cell electrodes to ensure their sufficient gas permeability, was noted.

Thus, it was shown that all oxide layers were nanoscale and did not contain defects in the form of delaminations, fractures or impurity inclusions that could negatively affect their functional properties.

**Figure 5:**
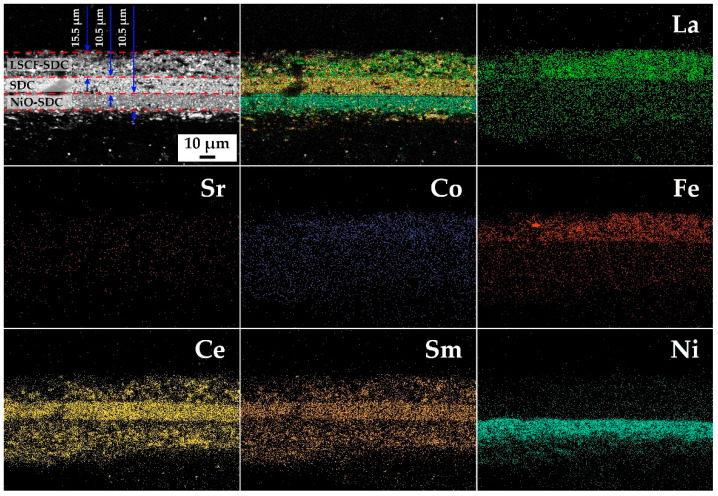
Microstructure of the cross-sectional area of the printed three-layer structure of the composition (NiO-SDC)/SDC/(LSCF-SDC), as well as chemical element distribution maps of its surface.

To examine the microstructural features of the (NiO-SDC)/SDC/(LSCF-SDC) three-layer structure formed on the surface of the Al_2_O_3_ substrate, a corresponding cross-section was fabricated and further analyzed using scanning electron microscopy (Figure 5). As can be seen in the micrographs obtained with the backscattered electron detector, the differences in the chemical compositions of the functional layers appeared in phase contrast, which allowed us to observe clear interfacial boundaries. For the NiO-SDC and LSCF-SDC layers, their composite structures and the uniform distribution of (CeO_2_)_0.80_(Sm_2_O_3_)_0.20_ particles in their volume were clearly visible. A color visualization (Cameo+ function) of the cross-section area of the three-layer oxide structure, which takes into account the results of the chemical element mapping on the studied surface, also clearly demonstrated the distinct boundaries between the separate layers and the absence of significant defects, and it also enabled a more accurate determination of the thickness of each layer, as well as confirming the compliance of light inclusions in the composition of the NiO-SDC and LSCF-SDC layers with (CeO_2_)_0.80_(Sm_2_O_3_)_0.20_ solid solution particles. The element distribution maps of the cross-sectional area confirm that the lower functional layer mainly contained nickel oxide and samarium-doped ceria distributed in its volume. The second layer contained cerium and samarium, which is consistent with the expected structure of the material. The third layer contained lanthanum, iron and cobalt (the strontium signal was weaker), as well as cerium and samarium. Thus, the chemical element mapping of the surface of the studied cross-section confirms the formation of a three-layer structure of the composition (NiO-SDC)/SDC/(LSCF-SDC) with a thickness of about 36.5 μm. The two lower layers (NiO-SDC and SDC) were the same thickness (10.5 μm), and the upper composite layer (LSCF-SDC) was about 15.5 μm thick. Taking into account that each of these layers of different compositions was formed via microextrusion printing in a single pass, in general, such similar thickness parameters can be considered an important advantage of the employed approach.

**Figure 6:**
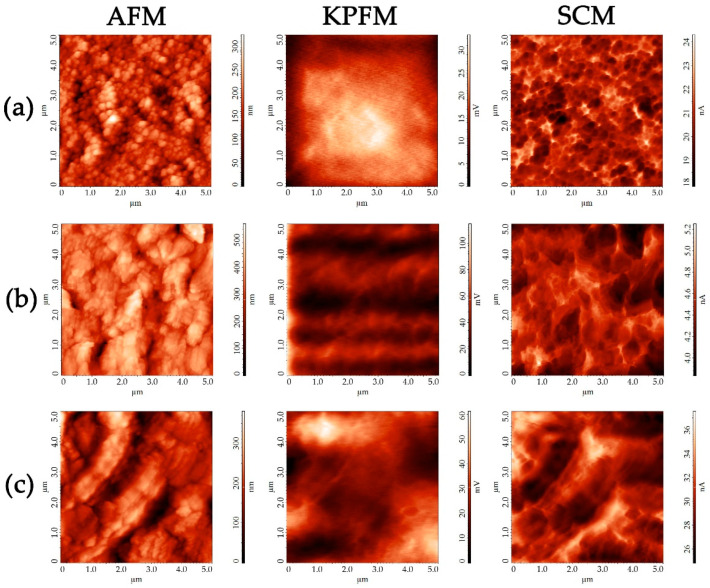
Topography of the coatings deposited sequentially on the Pt/Al_2_O_3_/Pt chip surface (left column), as well as surface potential (center) and capacity gradient (right) distribution maps for NiO-SDC (**a**), (NiO-SDC)/SDC (**b**) and (NiO-SDC)/SDC/(LSCF-SDC) layers (**c**).

The morphology (the size and shape of particles and roughness) and local electrophysical characteristics (the work function of the material surface, distribution of charge carriers and capacitance values of the “probe tip—surface of the material” capacitor) of the oxide layers of different compositions formed successively on the Pt/Al_2_O_3_/Pt chip were studied by means of several AFM techniques. It was found that, generally, the microstructural characteristics of the particles in the oxide coatings are in good agreement with the corresponding parameters of the nanopowders used. Thus, in the case of the NiO-SDC coatings, particle agglomerates of about 200 nm in size were preserved in its composition (Figure 6a). According to the AFM results, when the second layer ((CeO_2_)_0.80_(Sm_2_O_3_)_0.20_) was applied onto the NiO-SDC coating, the surface of the lower layer overlapped completely, which could be observed via a significant relief change: the surface was formed by elongated agglomerates from 200 nm to 2 μm long (Figure 6b). The oxide nanopowder particles used in the preparation of the corresponding functional inks had a similar microstructure. For the top layer of the (NiO-SDC)/SDC/(LSCF-SDC) structure, both the elongated agglomerates characteristic of samarium-doped ceria and the accumulation of less ordered nanoparticles characteristic of La_0.6_Sr_0.4_Co_0.2_Fe_0.8_O_3−δ_ oxide (Figure 6c) were observed. The roughness of the coatings varied in accordance with the microstructure features of a particular material. Thus, for the one-layer coating (NiO-SDC), the mean square roughness of an area of about 300 μm^2^ was 58 nm; for the two-layer coating ((NiO-SDC)/SDC), it was 92 nm; and for the three-layer coating ((NiO-SDC)/SDC/(LSCF-SDC)), it was 70 nm. The increased surface roughness of the SDC layer is probably related to its composition of hierarchically organized agglomerates larger in size than the particles of the first layer. Surface potential distribution maps were built during the surface scanning of the oxide layers in the KPFM mode. One could see that, in the case of the NiO-SDC layer, there was a uniform potential distribution, which is usually due to the relatively high electronic conductivity of the material, probably ensured by the nickel oxide (Figure 6, middle column). For the second oxide layer surface, the existence of areas with an increased potential was noted, which indicates a reduced electronic conductivity, leading to the appearance of local accumulations of small charges. When studying the third layer surface, there was a noticeable contrast between the LSCF and SDC agglomerates in terms of the surface potential and a slight difference in the value of the work function (4.87 eV for the (CeO_2_)_0.90_(Sm_2_O_3_)_0.20_ anisotropic agglomerates; 4.83 eV for the La_0.6_Sr_0.4_Co_0.2_Fe_0.8_O_3-δ_ particles). In general, the work function value of the studied material surface gradually decreased with each subsequent layer: for NiO-SDC, it was 5.02 eV; for the (NiO-SDC)/SDC two-layer coating, it was 4.93 eV; and for the three-layer structure of composition (NiO-SDC)/SDC/(LSCF-SDC), it was 4.83–4.87 eV (depending on the coating region). Concerning the SCM maps of the capacitance gradient distribution over the oxide layer surface (Figure 6, right column), one could observe lighter areas on the material grain boundaries in all cases. These are related to increased capacitance changes in the boundaries during the formation of depletion layers due to the higher concentration of charge carriers on the boundaries. This, in turn, indicates the grain-boundary mechanism of electronic conductivity in the oxide layers.

**Figure 7:**
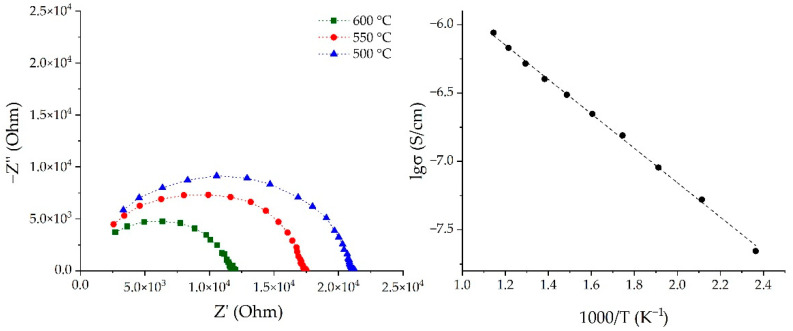
Frequency dependences of the impedance of the (NiO-SDC)/SDC/(LSCF-SDC) three-layer structure (**left**) and the temperature dependence of its specific conductivity (**right**).

The electrical conductivity of the (NiO-SDC)/SDC/(LSCF-SDC) three-layer nanostructure was studied using electrochemical impedance spectroscopy. Figure 7a shows typical impedance hodographs of the sample under study—it can be seen that they have the form of semicircles slightly extended along the abscissa axis. The intercept of the impedance arc with the real axis at low frequencies represents the total resistance of the three-layer structure under consideration. It was found that the diameter of the semicircles decreased with an increase in the temperature, since the charge transfer process is more active, which leads to a decrease in the resistance of the sample. Using impedance spectroscopy data, the temperature dependence of the total conductivity of the material was estimated, and it was determined that the conductivity increased linearly by 1.5 orders of magnitude as the temperature was increased from 150 to 600 °C (Figure 7b). It is known that a high-density electrolyte layer is preferable when creating conventional SOFCs, since it helps to reduce the resistance of the fuel cell. In our case, the conductivity of the printed three-layer structure had a rather low value, but a significant increase in this parameter can be achieved by the additional sintering of the components at higher temperatures. One of the most important results of this study is the demonstration of the potential of the developed technology for the microextrusion printing of miniaturized multilayer hierarchically organized conducting nanostructures of a given geometry, which is promising for the evolution of SOFC miniaturization approaches.

## 4. Conclusions

In the course of this study, NiO, La_0.6_Sr_0.4_Co_0.2_Fe_0.8_O_3–δ_ (LSCF) and (CeO_2_)_0.8_(Sm_2_O_3_)_0.2_ (SDC) nanopowders characterized by different microstructures—0D, 1D and 2D, respectively—were obtained using hydrothermal and glycol–citrate methods. It was shown that the nickel(II) oxide powder consisted of aggregated spherical nanoparticles sized 25 ± 3 nm. The LSCF was composed of two-dimensional formations with lateral sizes of up to tens of micrometers and a thickness of about 120 nm, with a bimodal particle size distribution—the films consisted mainly of faceted grains of 120 ± 15 nm with finer particles (about 20 ± 4 nm) on their surface. In the case of the SDC sample, the powder had a multilevel hierarchical self-organization of particles—nanoscale isotropic particles were ordered into one-dimensional structures about 5 µm long and 200–300 nm wide, combined into bundles, which, in turn, were coordinated at an angle of about 60° to each other to form six-point structures.

Using functional inks based on the obtained oxide nanopowders, the NiO-SDC, SDC and LSCF-SDC coatings were sequentially applied to the surfaces of various substrates using microextrusion printing, resulting in three-layer structures of the composition (NiO-SDC)/SDC/(LSCF-SDC).

According to the XRD data, a chemical interaction between the substrates and components of the formed functional layers of different compositions did not occur during additional heat treatment. NiO retained the crystal structure of the halite type (average CSR size was 23 ± 2 nm), SDC retained a structure of the fluorite type (11 ± 1 nm), and LSCF retained a structure of the perovskite type (28 ± 3 nm). Based on the SEM and AFM data, the microstructural features of the obtained oxide layers were inherited from the corresponding powders; the materials were nanoscale and contained no defects in the form of delaminations, fractures or impurity inclusions; and for the NiO-SDC and LSCF-SDC layers, the composite structures were confirmed. When studying the cross-sectional area of the three-layer structure of (NiO-SDC)/SDC/(LSCF-SDC) composition, element distribution maps were constructed, which confirmed the target compositions of all functional layers and distinct interfacial boundaries. The resulting thickness of the formed multilayer hierarchically organized structure was about 36.5 μm. The thicknesses of the two lower layers (NiO-SDC and SDC) were the same (10.5 μm), and the upper composite layer (LSCF-SDC) was about 15.5 μm thick. The resulting thickness of the formed multilayer hierarchically organized structure was about 36.5 μm. The thicknesses of the two lower layers (NiO-SDC and SDC) were the same (10.5 μm), and the upper composite layer (LSCF-SDC) was about 15.5 μm thick. Thus, microextrusion printing made it possible to achieve similar thickness values for the layers of different compositions in one pass in each case.

The SCM maps of the capacitance gradient distribution over the surface of the sequentially deposited oxide layers demonstrated that a shift in the charge carrier density to the boundaries between the grains occurred in all cases. Using KPFM, it was shown that the work function value of the studied material surface gradually decreased with the application of each successive layer: it was 5.02 eV for NiO-SDC, 4.93 eV for the two-layer coating (NiO-SDC)/SDC and 4.83–4.87 eV (depending on the coating region) for the (NiO-SDC)/SDC/(LSCF-SDC) three-layer structure.

For the printed three-layer nanostructure of composition (NiO-SDC)/SDC/(LSCF-SDC), the temperature dependence of conductivity was determined, and it was shown that the conductivity increased linearly by 1.5 orders of magnitude when the temperature was increased from 150 to 600 °C.

One of the key results of this study is the demonstration of the capability of the developed technology for the microextrusion printing of miniaturized multilayer hierarchically organized conducting nanostructures of a given geometry, which is promising for the development of SOFC miniaturization approaches.

## Data Availability

Not applicable.
